# A Systematic Review and Meta-Analysis Study to Investigate the Prevalence of *Helicobacter pylori* and the Sensitivity of its Diagnostic Methods in Iran

**DOI:** 10.5812/ircmj.12581

**Published:** 2014-06-05

**Authors:** Fatemeh Sayehmiri, Zahra Darvishi, Kourosh Sayehmiri, Setareh Soroush, Mohammad Emaneini, Raffaele Zarrilli, Morovat Taherikalani

**Affiliations:** 1 Student Research Commitee, Ilam University of Medical Sciences, Ilam, IR Iran; 2 Prevention of Psychosocial Injuries Research Center, Ilam University of Medical Sciences, Ilam, IR Iran; 3 Department of Microbiology, School of Medicine, Tehran University of Medical Sciences, Tehran, IR Iran; 4 Clinical Microbiology Research Center, Ilam University of Medical Sciences, Ilam, IR Iran; 5 Department of Public Health, University of Naples Federico II, Naples, Italy

**Keywords:** *Helicobacter pylori*, Prevalence, Meta-analysis, Iran

## Abstract

**Context:**

*Helicobacter pylori* is the most common infecting organism affecting humans, being almost half the population infected. The aim of this study was to find out the epidemiological features and the sensitivity of diagnostic methods of *Helicobacter pylori* infection in Iran, which can provide the logic of developing preventive approaches to control *Helicobacter pylori* infections and the associated diseases.

**Evidence Acquisition:**

By reviewing the databases of SID, MAGIRAN, SCOPUS, MEDLIB, Google, ISI and PUBMED, a total of 30 papers, published from 1994 to 2011, were extracted. Summary prevalence and 95% confidence intervals (95% CI) were calculated using random-effects model. Statistical analyses were performed using STATA Ver.11.

**Results:**

Among the 30 studies evaluated, the prevalence rate of *Helicobacter pylori* infections in Iran was estimated as 50.7 % (95% CI: 44.4-56.9%). When the sensitivity of diagnostic methods was evaluated, ELISA, with an accuracy rate of 52.3% (95% CI: 43.8 to 60.8%) was found the most accurate diagnostic method available. The highest and the least *Helicobacter pylori* prevalence were 19.2% in Tehran and 74.27% in Mazandran respectively.

**Conclusions:**

About half the population in Iran is infected with *Helicobacter pylori*, the pollution in different areas and preventive strategies should be carried out to control this infection. The prevalence rate of *Helicobacter pylori* infections in Iran showed only little changes during the years 1994 to 2011.

## 1. Context

Almost half the population in the world is infected by *H. pylori* which is the most common infecting organism affecting humans (1, 2). Since the first experience of Helicobacter culture in 2004 by Marshall and Warren, a considerable amount of information has been acquired on clinical aspects of this infection (3). Helicobacter pylori is tightly associated with gastro-intestinal disorders, some important consequences of which can be chronic gastritis, peptic ulcer leading to gastric cancer, indigestion and non-ulcer dyspepsia (4-9).

In developing countries, the prevalence of *H. pylori* positive serology during childhood is higher than that in developed countries. This is vice versa during adulthood (10, 11). In a Meta-analysis study, the prevalence of *H. pylori* infection in China was reported as 58.07% (12). In another Meta-analysis study, the association between *H. pylori* infection and the risk of esophageal cancer (EAC) has been suggested (13). Also, the association between *H. pylori* infection in diabetic and non-diabetic individuals with dyspepsia was assessed. This study showed that the prevalence of *H. pylori* infection was considerably higher in diabetic patients compared to non-diabetic individuals (P = 0.001). According to these results, diabetes Mellitus is one of the risk factors that need attention in the evaluation of *H. pylori* infections among diabetic patients with dyspepsia (14).

The prevalence of *H. pylori* infection was 58% among residents of two villages in Northern Italy, considerably higher than the 34% observed in an earlier similar study of adults in northern Swedish communities (mean age = 52 years). A prevalence of 60% or more was reported for groups in Albania, Egypt, Iran, Turkey, and China (15). The prevalence of *H. pylori* infection was 3.0% among index children at age 4 and maternal infection was the only risk factor at multivariate analysis (16).

One of the main objectives of Meta-analysis studies, which are a combination of different studies, is to reduce the difference between parameters as a result of an increase in the number of studies involved in the process of analysis and also to reduce the confidence level of these measures/figures.

The aim of this study was to investigate the studies carried out on the prevalence of *H. pylori* infection in Iran and to estimate the prevalence rate of this infection according to diagnostic methods, age group of individuals infected, gender, infection age and additional parameters.

## 2. Evidence Acquisition

This study was a systematic review and a Meta-analysis study on the prevalence of *Helicobacter* in Iran. The findings of this research were based on the papers published in internal and external journals. The papers were selected from the databases of SID, MAGIRAN, SCOPUS, MEDLIB, Google, ISI and PUBMED. Searching of the articles was carried out using the key words: Helicobacter, prevalence, and a combination of these words. To decrease bias two authors (Sayehmiri F, Darvishi Z) did search, selection of papers and extracting data of articles independently. First, all the articles related to Helicobacter pylori in Iran were collected. At this stage, the articles possessing the relevant key words in their title or abstract were entered into the list, excluding all the other articles not relevant to the prevalence of *H. pylori.* A check list of necessary information was prepared that included: title of the article, location of the study, location of sampling, sample volume, the methods of assessing infection, the mean age of individuals infected by *H. pylori* and the total prevalence of *H. pylori*.

**Figure 1.**
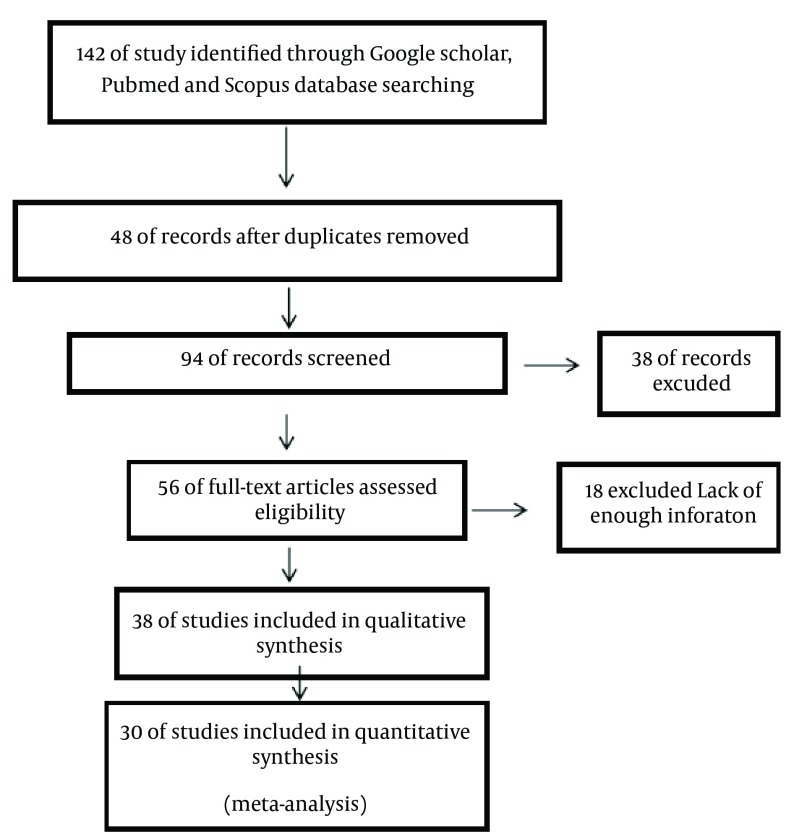
Study Flowchart

According to the protocol, a total of 142 articles that were carried out from 1994 to 2011 were analyzed during the primary research. The abstracts of 94 articles, out of the 142, were then analyzed and a complete review of 45 of these papers, that included the basic information required, was then carried out. Due to unavailability of the prevalence rates, some articles were omitted and a total of 30 articles were finally entered into the process of analysis (17-46) (Figure 1 the study flowchart).

### 2.1. Statistical Analysis

The variance of each study was calculated according to the binomial distribution. The studies were combined together according to sample size and variance. Because of the study heterogeneity, random-effect model was used to combine the studies. To assess the heterogeneity of the studies, Cochrane Q test and the I^2^ index were used. P-value of less than 5% was considered as significant in the heterogeneity test. To investigate the relationship between years of study and sample size meta-regression model was used.

Furthermore, considering the type of data analyzed, which were all prevalence rates, there was no need to determine the publication bias and to draw a funnel plot. The data were analyzed using STATA version 11.2.

## 3. Results

**Figure 2.**
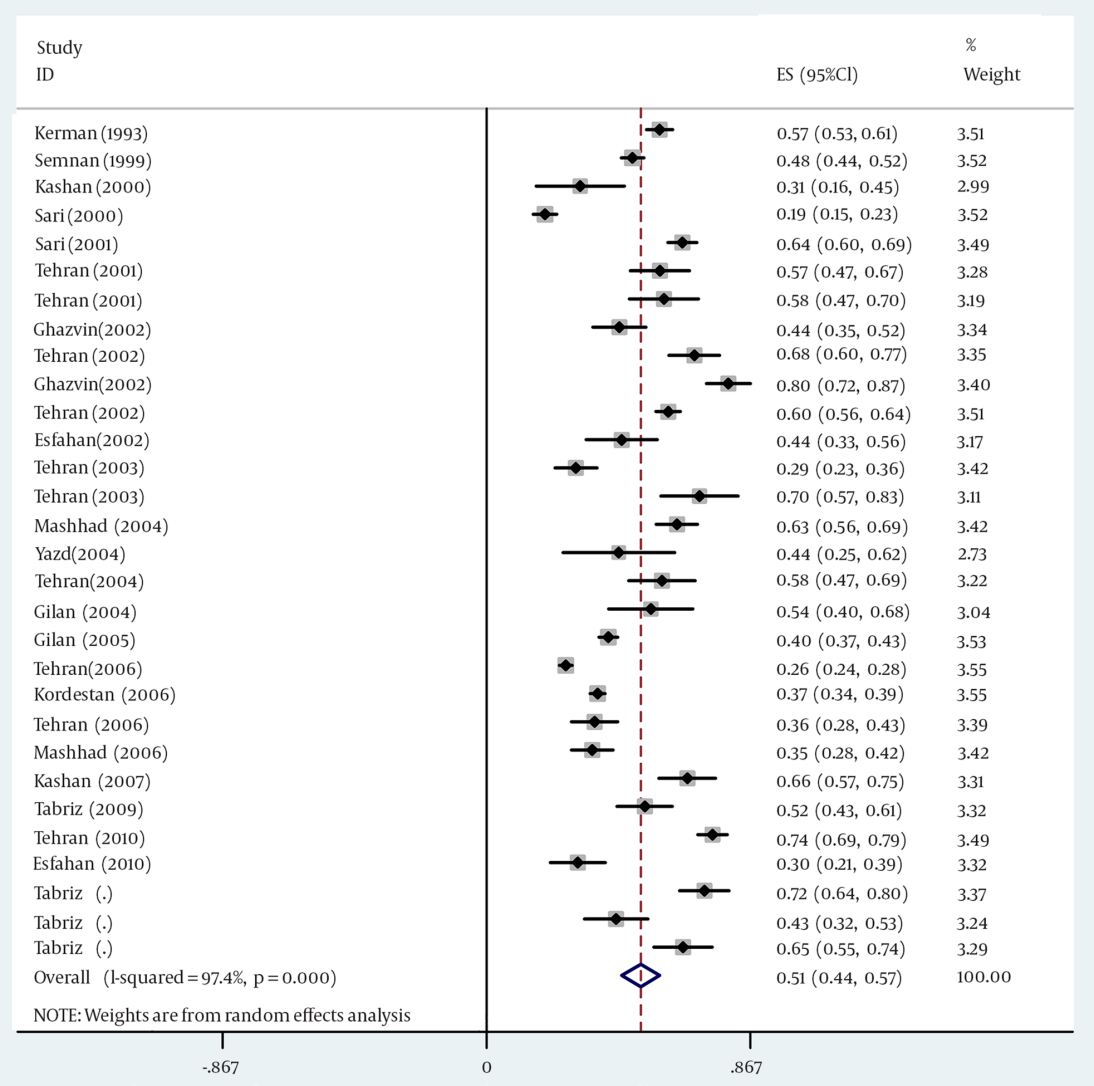
The Prevalence Rate of H. pylori Infection and its 95% Confidence Interval According to the year and the location of studies assessed. The midpoint of each line illustrates the prevalence rate estimated in each study. The diamond sign shows the prevalence rate throughout the country for all the studies.

In the 30 papers assessed, the prevalence rate of Helicobacter pylori infection in Iran was estimated as 50.7% (95% CI: 44.4-56.9%) (Figure 2). The lowest prevalence rate (19.2%) was related to a study in Mazandaran province, the city of Sari and the highest prevalence rate (74.27%) were reported in a study in Tehran province, the city of Tehran. Details of the papers analyzed are found in Table 1.

**Table 1.** Summary of Included Studies Evaluating the Prevalence Rate of *H. pylori* in Iran

City	year	Age,y	Sample Size	Diagnostic Method	Prevalence	95% CI		References
**Kashan**	2000	4.1 ± 9.9	39	Urease	30.8	45.3	16.3	Taghavi et al. (30)
**Sari**	2000	7-18	400	-	19.2	23.1	15.3	Tergar Fakheri et al. (39)
**Semnan**	1999		700	Elisa	48	57.1	44.3	Moradi et al . (22)
**Ghazvin**	2002	54.4	125	Urease	43.7	52.4	35	Hajagamohammadi et al. (17)
**Sari**	2001		394	Elisa	64.2	69.1	59.7	Baba mahmoodi et al. (38)
**Khorasan razavi**	2004	12-75	195	Urease	62.56	69.4	55.8	Nakhaei Moghadam et al. (40)
**Tehran**	2002	30.65	120	Urease	68.3	76.6	60	Faghihi et al. (23)
**Yazd**	2004	30-65	28	-	43.5	61.9	25.1	Benesh et al. (41)
**Ghazvin**	2002	10-60	240	Elisa	2000.5	86.7	1993.3	Sheikholslami et al. (18)
**Tabriz**	2009	3.21 ± 8.38	116	Serology	Children 60.3, Mothers 57.8, Fathers 37.9	61.1	42.9	Rafiei et al. (31)
**Kerman**	1993	57.05	513	Urease	56.9	61.2	52.6	Zojajy (19)
**Tehran**	2002	8.8 ± 48	576	Endoscopy	59.7	63.7	55.7	Yasseri (24)
**Tehran**	2004	2.2 ± 24	137	Elisa	65.57	75.3	56.7	Sharifian et al. (32)
**Tehran**	2004-2006	6-12	1665	Urease	26	28.1	23.9	Zamani et al. (25)
**Tehran**	2003	-	170	Histopathology	29.4	36.2	22.6	Barati et al. (26)
**Tabriz**	-	-	120	-	71.6	2000.7	63.5	Rahnama et al. (43)
**Tabriz**	-	-	86	-	42.6	53.1	32.1	Rafeey et al. (44)
**Tabriz**	2003	0.03 ± 8.3	96	-	64.6	74.2	55	Modaresi et al. (45)
**Kurdistan**	2006	14.91 ± 15.32	1503	Elisa	36.5	38.9	34.1	Yazdanpanah et al. (33)
**Tehran**	2006	39	152	-	35.52	43.1	27.9	Shokohizadeh et al. (46)
**Tehran**	2010	43.9	311	-	74.27	2000.1	69.4	Neshandar et al. (34)
**Kashan**	2007	17.13 ± 43.3	100	Elisa	66	75.3	56.7	Arj et al. (20)
**Tehran**	2003	11.67 ± 39.68	50	Urease, IgG, Histology	70	2003.7	57.3	Baghaei et al. (42)
**Isfahan**	2010	16-62	100	PCR	30	39	21	Salehi et al. (27)
**Guilan**	2004	11.30 ± 32.38	50	Elisa	54	67.8	40.2	Ghanei et al. (28)
**Tehran**	2001		100	Urease, Pathology	57	66.7	47.3	Gottaslou et al. (29)
**Guilan**	2005	7-11	961	Elisa	40	43.1	36.9	Mansor et al. (35)
**Tehran**	2001	48.3	1993	Citology	58.3	69.7	46.9	Vahidi et al. (36)
**Isfahan**	2002		70	Elisa	44.44	56.1	32.8	Sanei et al. (21)
**Khorasan razavi**	2006	4.77 ± 23.50	187	Elisa	34.7	41.5	27.9	Dolatian et al. (37)
-	-	-	-	-	50.7	56.9	44.4	-

In one classification, analysis was carried out based on the diagnostic methods. The sensitivity level of ELISA, according to this analysis, was estimated as 52.3% (95% CI: 43.8-60.8), the sensitivity of the urease method as 51.9% (95% CI: 36.9-66.9) and other diagnostic methods including touch cytology, histopathology as 45.8% (95% CI: 31.3-60.4). These results show that ELISA and urease are relatively more accurate methods to diagnose *H. pylori* infection (Table 2).

In another classification, the individuals under assay were divided into the two groups of symptomatic and non-symptomatic individuals. The prevalence rate of infection among individuals with digestive disease symptoms, with a sample size of 1873 individuals, was estimated as 47.6% (95% CI: 56.1-39.2) and among healthy individuals, with a sample size of 7131 individuals, as 53.1% (95% CI: 45.4-60.8). Based on gender, the prevalence rate was estimated as 37.7% (95% CI: 95% CI: 27.5-48) in females and 35.7% (95% CI: 19.6-51.7) in males. In analysis based on the age groups, the prevalence rate among the age group below 20 years was estimated as 38.3% (95% CI: 28.1-48.5), among the age group between 20-40 years as 50.3 % (95% CI: 39.5-61) and among the age group above 40 as 60.1% (95% CI: 52.4-67.8). In this analysis, a significant difference was found among the three age groups which can be evidence to the conclusion that the rate of *H. pylori* infection increases as the age increases (Table 3).

**Table 2.** Prevalence Rate of *H. pylori* Infection in Iran According to Different Diagnostic Methods

Diagnostic Methods	Study, No.	*H. pylori*, %, 95% CI
**Total Recognize group**	30	50.7 (44.4-56.9)
**ELISA**	10	52.3 (43.8-60.8)
**Urease test**	8	51.9 (36.9-66.9)
**Other methods**	5	45.8 (31.3-60.4)

**Table 3.** Prevalence Rate of *H. pylori* Infection in Iran According to Health Status, Age and Gender

	Study, No.	*H. pylori*, %, 95% CI
**Total**	30	50.7 (44.4-56.9)
**Sex**	-	-
Women	5	37.7 (27.5-48)
Men	5	35.7 (19.6-51.7)
**Population**	-	-
Patient	17	47.6 (39.2-56.1)
Healthy	13	53.1 (45.4-60.8)
**Age, y**	-	38.3 (28.1-48.5)
≤ 20	6	50.3 (39.5-61)
20-40	7	60.1 (52.4-67.8)
> 40	6	-

**Figure 3.**
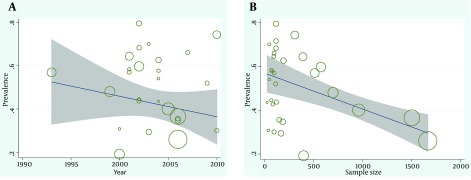
*H. pylori* Prevalence Rate According to the Year of Study and Sample Size The fitted line show meta-regression line.

The Figure 3 shows the relationship between the prevalence of *H. pylori* infection and year of study, and sample size using meta regression models. In Figure 3 A the negative slope of the meta regression line showed that the prevalence of *H. pylori* infection in Iran has had a decreasing trend with a slow slop but it was not significant. In Figure 3 B the relation between sample size was compared to the prevalence rate of *H. pylori* infection and according to this figure there isn’t a significant relation between the sample size and prevalence (P = 0.06). At the following figure the circles show the weight of studies and it seems that studies with greater sample sizes are more prevalent and vice versa (Figure 3). In the Table 4, a summary of data related to meta-regression curves are shown.

**Table 4.** The Unadjusted and Adjusted Regression Coefficients and Corresponding P Values for Correlation Between Potential Influential Factors and *H. pylori* Prevalence Using meta-Regression Models

Factors	Regression Coefficients	P
**Year of data collection**	-	-
Unadjusted	-0.00332003004	0.707
Adjusted	0.0086586	0.21999
**Age group**	-	-
Unadjusted	0.1077322	0.017
Adjusted	0.1222877	0.008
**Sample size**	-	-
Unadjusted	-0.0001338	0.060
Adjusted	-0.000919	0.159

**Figure 4.**
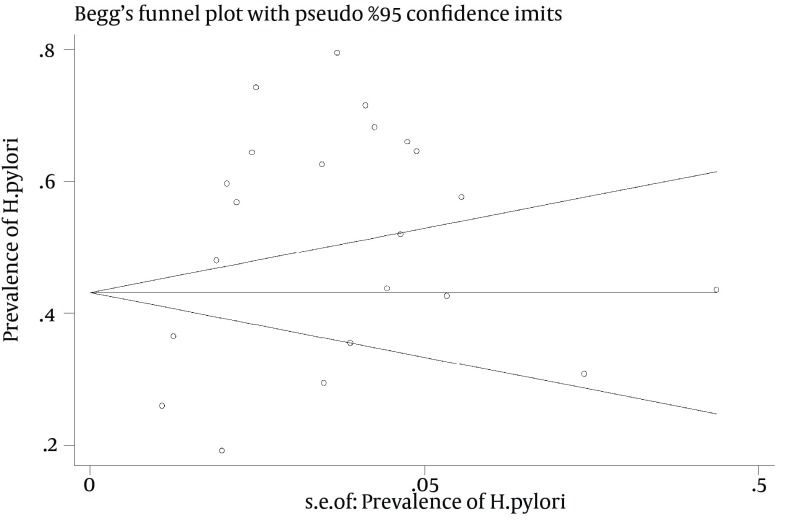
Funnel Plot to Assess Publication Bias

To assess publication bias we used funnel plot (Figure 4) that it showed there was a significant publication bias (P = 0.016). In another classification, the analysis was carried out based on the city of the study, according to which the highest prevalence rate (61.7%), with a sample size of 245 individuals, was related to Ghazvin province (95% CI: 26.6-96.8) and the lowest prevalence rate (36.5%), with a sample size of 73 individuals, was related to Kurdistan province (95% CI: 38.9-34.1) (Table 5).

**Table 5.** Prevalence Rate of *H. pylori* in Iran According to City

City	Random Effects OR, 95% CI	I^2^, %	Sample Size	Study, No.
**Kashan**	48.9 (14.4-2004.3)	93.8	139	2
**Sari**	41.8 (-0.025-0.86)	99.5	20004	2
**Semnan**	48 (44.3-51.7)	-	700	1
**Ghazvin**	61.7 (26.6-96.8)	97.4	245	2
**Khorasan Razavi**	48.6 (21.3-75.9)	96.9	32003	2
**Tehran**	53.5 (39.1-67.8)	98.4	3295	10
**Yazd**	43.5 (25.1-61.9)	-	28	1
**Tabriz**	57.9 (45.3-70.5)	86.6	418	4
**Kerman**	56.9 (52.6-61.2)	-	513	1
**Kurdistan**	36.5 (34.1-38.9)	73	1503	1
**Isfahan**	36.7 (22.6-50.8)	73.4	170	2
**Guilan**	45.3 (32-58.6)	97.4	1011	2
**-**	50.7 (44.4-56.9)	-	9198	30

## 4. Discussion

A data containing 9198 *Helicobacter pylori*-infected individuals were included in the current study. A total of 30 papers were entered into the final Meta-analysis. I^2^ statistics showed a high variation among studies results (I^2^ = 97.4, P = 0.000). In three studies, including those carried out in Gilan, Sari and Qazvin, sampling had been carried out in villages as well as in cities (17, 33, 38). The prevalence rate of *H. pylori* was calculated for all the individuals in all the age groups, based on the status of the individuals, gender and age groups.

In our review, random effect models were used for meta-analysis, considering the possibility of significant heterogeneity between studies which was tested with the Q test.

According to this model, it is assumed that individual studies are estimating different treatment effect. Another study performed by the research team of Eslami and colleagues focused on the association between *H. pylori* infection and the risk of esophageal cancer (EAC) (13). In a Meta-analysis study carried out in China in 2003, the pooled prevalence rate of *H. pylori* infection in Chinese population was estimated as 58.07%, 50% of which was related to the age group of 10-20 years. This Meta-analysis has been extracted from the articles published during the years 1990-2002. In this study, infection with *H. pylori* has been reported to be a risk factor for the development of digestive system disorders (12). In the study of Kashani and his colleagues, the co-existence of *H. pylori* with non-ulcer dyspepsia has been assessed in different groups of patients. In this study, no significant correlation was found between *H. pylori* infections and dyspepsia (23). In the study of Zamani and colleagues, there was a significant difference in the prevalence rate of *H. pylori* infection among males and females (25). In another study in Tabriz city, the relationship between the prevalence rate of *H. pylori* infection among children and their parents was evaluated. This study showed that the infection of parents, especially mothers, has a key role in the transmission of infection into their children and that aging, due to increased environmental exposure, would increase the risk of *H. pylori* infection. According to the results of this study, Father’s age and job and mother’s serology and literacy are important factors in the infection of the child with *H. pylori* (10). In the study of Sharifian and his colleagues, carried out on the students of medicine and dentistry, it was found that the prevalence rate of *H. pylori* infection among individuals with digestive illness was lower and that the prevalence rate of *H. pylori* does not increase with an increase in the duration of dental work (32).

In a study carried out in Sari in 132001, a significant correlation was reported between the prevalence rate of infection and home ownership and the family size (38). In the study by Azevedo et al. the major risk factors that have been emphasized were the socioeconomic indicators (15).

Among the limitations of the current study, the following can be mentioned:

1. There was not same method to measure variables and diagnostic tests among all studies as well as,
2. The majority of the studies were carried out in a preselected population (blood donors, individuals referring to health care centers and etc.).
3. In most studies, nutritional status and life style were not considered as influencing factors.
4. The range of age groups was considerably wide among different studies.

This study aimed to determine the prevalence rate of *H. pylori* infection in Iran. Almost half the population in Iran is infected by *H. pylori*, the prevalence rate being different among different parts of the country. According to the findings of this study, it is suggested that, to prevent the spread of *H. pylori*, comprehensive health programs should be scheduled throughout the country. According to the results of this study, the risk of *H. pylori* infection significantly increases with age, and so, it is recommended that preventive procedures are more seriously followed for high risk age groups. It is also recommended that the ELISA and urease tests are used in diagnostic laboratories to increase the accuracy and perception of diagnostic methods.

Funnel plot (Figure 4) showed the effects of publication bias was significant in this study. Publication bias is very important in randomized clinical trials and case control studies, but it is not important in the meta-analysis that estimates prevalence publication bias.
